# Comparison of different methods to assess the distribution of alien plants along the road network and use of Google Street View panoramas interpretation in Sicily (Italy) as a case study

**DOI:** 10.3897/BDJ.9.e66013

**Published:** 2021-05-27

**Authors:** Giulio Barone, Gianniantonio Domina, Emilio Di Gristina

**Affiliations:** 1 Department of Agricultural, Food and Forest Sciences, University of Palermo, Palermo, Italy Department of Agricultural, Food and Forest Sciences, University of Palermo Palermo Italy

**Keywords:** road ecology, roadside, alien invasive plants, remote sensing

## Abstract

The survey by foot in the field is compared to the survey from a car, the photo-interpretation of Google Street View (GSV) panoramas continuously and at intervals of 1.5 km and the photo-interpretation of Google Earth aerial images on a 10 km stretch of road in Sicily. The survey by foot was used as reference for the other methods. The interpretation of continuous GSV panoramas gave similar results as the assessment by car in terms of the number of species identified and their location, but with lower cost. The interpretation online of aerial photos allowed the identification of a limited number of taxa, but gave a good localisation for them. Interpretation of GSV panoramas, each of 1.5 km, allowed the recognition of twice as many taxa as the interpretation of aerial photos and taking half the time, but did not allow a complete localisation.

None of these methods alone seems sufficient to carry out a complete survey. A mixture of different techniques, which may vary according to the available resources and the goal to be achieved, seems to be the best compromise.

To further test the capabilities of the survey using the interpretation of GSV panoramas every 1.5 km along the roads, we proceeded to study the alien plants along 3500 km of the road network on the island of Sicily. This survey identified only 10% of the known species for the region, but allowed us to trace the distribution of invasive species whose distribution is currently poorly recorded.

## Introduction

Alien plants are plants that have been transported by humans intentionally or accidentally outside their area of origin (Pyšek et al. 2004). Some of the species that successfully establish in an area where they have been introduced spread very rapidly with serious damage to the native species and ecosystems of that place; these are called “invasives”. Authorities and researchers, therefore, have a great interest in studying distribution and dispersion of aliens in different habitats. Scientific research on alien species in Italy, in recent years, has not been limited only to floristic notes, but has increasingly deepened the methodologies of analysis, the ecological impacts of aliens and their modes of dispersal (see, for example, Stinca et al. 2013, Domina et al. 2018b, Lozano et al. 2019, Lazzaro et al. 2020, Viciani et al. 2020). It is now well established that roads promote the dispersion of alien plants and have ecological impacts on many habitats modifying the microclimatic conditions of the area they pass through and the immediate vicinity (Wilcox 1989). In fact, roads may act as corridors for seed dispersal due to continue anthropogenic disturbance (Taylor et al. 2012, von der Lippe et al. 2013). Roadsides provide favourable conditions for the establishment and spread of alien invasive plants due to recurrent disturbance, more light and water (Parendes and Jones 2000, Christen and Matlack 2006). Some plant species also invade nearby areas (Hansen and Clevenger 2005).

There are already some examples of monitoring the presence of alien species along road networks (e.g. Tikka et al. 2001, Brundu et al. 2003, Gelbard and Belnap 2003, Christen and Matlack 2006, Barbosa et al. 2010, Kotowska et al. 2021). Traditionally, surveys of flora and vegetation are carried out on foot in test areas (Rew and Pokorny 2006, Brundu et al. 2011). Remote sensing techniques have become a very useful tool for this type of analysis, allowing us to considerably enlarge the investigated areas (Mararakanye et al. 2017). Road surveys conducted by car are a common method for monitoring and assessing the distribution of alien invasive plants (McAvoy et al. 2017). The results of studies along roadsides may be generalised for the assessment of alien species over larger areas, but should be considered incomplete because they are focused on a single habitat.

Car surveys have the advantage of covering larger areas in shorter periods with fewer resources, when compared to on-foot surveys (Brundu et al. 2003, Shuster et al. 2005, Catry et al. 2015).

Currently on the internet, there are freely-available sources of images at ground level from which it is possible to obtain information on the plants present. The most famous and complete of all is certainly Google Street View (hereafter GSV). This is a technology featured in Google Maps and Google Earth that provides interactive panoramas, acquired with a complex camera system placed on moving cars, from positions along many streets in the world. Recently, it has been suggested as a cost-effective alternative for habitat assessment and the detection of alien plants along roads (Shuster et al. 2005, Barbosa et al. 2010, Olea and Mateo-Tomás 2013, Visser et al. 2013, Deus et al. 2016).

In this study, we wanted to extensively test the potential of the analysis of GSV images on different habitats, to know whether this method is user-friendly, reliable and cost-effective. This method was compared with the survey on foot, by car and by aerial view. Sicily was a perfect test area because it is well documented from a floristic point of view and able to offer considerable environmental variations within a few tens of kilometres.

## Material and methods

Sicily is the largest Mediterranean island located at the centre of the Mediterranean Basin separated from the rest of Italy by a 3 km strait. It has an approximately triangular shape and is surrounded by 14 islands that are inhabited all year round. The coordinates of the extreme points of the main island are respectively: 38 ° 18'5 "N for the northernmost point; 36 ° 38'48" N for the southern extreme; 12 ° 25'28 "E for the westernmost point and 15 ° 39'8" E for the easternmost point. Sicily is a predominantly hilly region (61.4% of the Island), while 24.5% is mountainous and the remaining 14.1% is flat. Its highest mountain is Mount Etna at 3,329 m. Sicilian rivers are all limited in flow rate and length (Sestini 1957). The only natural reservoir on the Island is the Pergusa Lake; the other reservoirs are all artificial with a more or less minimal flow. For a more detailed geographical description of the Island, see Gianguzzi and Bazan (2019). The Island has a total area of 25,711 km^2^ and a population of 4,969,147 inhabitants with a population density of 190/km^2^. The average annual temperature along the coast and in the plains that go inland is between 18 and 19°C; in the lower hills, between 17 and 19°C; in the upper hills, between 14 and 17°C; above 1000 m a.s.l., between 10 and 13°C. Only in a small portion at the top of the Etna mountain, the temperature goes down to 2°C. Average annual rainfall varies between 300 and 500 mm along the eastern part of the southern coastal belt and in the plain of Catania; between 500 and 700 mm along the western part of coastal belt and in the hilly interior; between 700 and 1000 mm on the mountainous chains of the Peloritani, Nebrodi, Madonie and Mounts of Palermo; values between 1000 and 1600 mm are recorded in the cacuminal belt of the Madonie, Nebrodi and Etna mountains. For a complete climatological classification of the Island, see Drago (2005).

The presence of humans on the Island dates back 14,000 years ago. The Island has always been used for agriculture. Urban development and the great expansion of the road system on the Island began around 1960 (Parrinello 2013). The road network of Sicily has been estimated at 18,315 km (Nobile and Zacchi 2019). Roads can be divided into three main categories: Highways, Main roads and Local roads. Highways in Sicily are about 24 m wide and have paved banks at the edges that are subjected to annual mowing by mechanical means. Main roads are about 14 m wide and have often unpaved banks that are subject to annual removal of natural vegetation by herbicides or by mechanical means. Local roads can be about 7 m wide or less and they may or may not be equipped with banks. The management of spontaneous vegetation along local roads is varied in terms of methods and times. The measures of the roads in Italy are established by Legislative Decree no. 285 of 30 April 1992. Their management is entrusted to the managing body of the single stretch of road.

### Data collection and analysis

Two analyses were conducted:

#### Comparison between different survey methods

A comparison was done on a 10 km stretch of coastal main road, between the reliefs on foot, by car, continuous interpretation of GSV panoramas, interpretation of GSV panoramas, each of 1.5 km and manual interpreting of Google Earth aerial photographs.

The area under investigation includes the roadway and 5 m on each side of the road no. 113 between Buonfornello (37.974130°N 13.825974°E, 8 m a.s.l.) and the Municipality of Lascari (38.011472°N 13.931456°E, 14 m a.s.l.) in the Province of Palermo.

**Figure 1.**
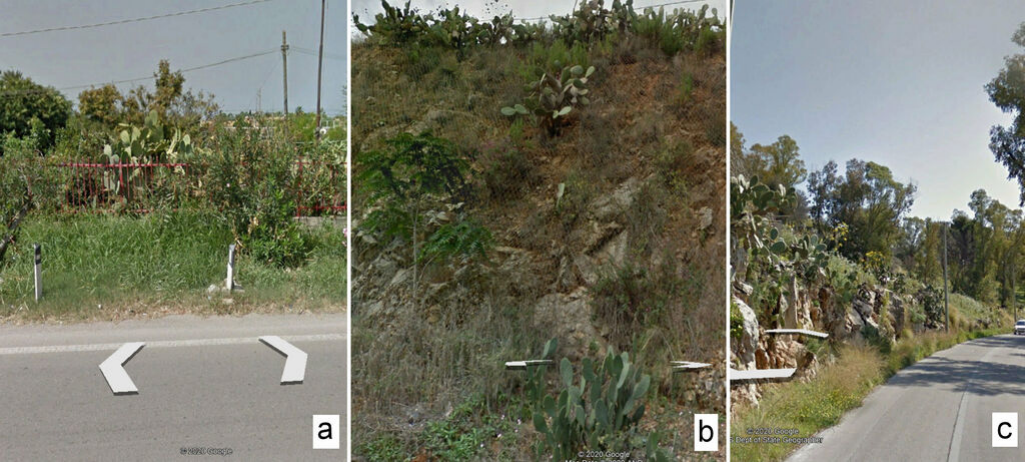
*Opuntia ficus-indica* in Google Street View shots: **a.** cultivated; **b.** cultivated with spontaneous regeneration; **c.** spontaneous.

The reliefs on foot and by car were done in September 2020; the photographic shots of the same stretch of road date back to September 2018; the aerial photos available on Google Earth date back to August 2019. The survey by car was carried out before the one on foot so that the operator was not influenced by the results of the more complete survey. The car ran around 35-40 km/h. The photo interpretations of GSV and Google Earth images were undertaken in September 2020, before the surveys in the field. Interpretation of GSV panoramas, each of 1.5 km, was done in seven points. It was decided to consider the survey on foot as a benchmark and to verify the difference in results of the other techniques with respect to this one. The measurements with the other methods were repeated five times and the expected loss analysis was performed [Expected_loss = (x_med-target)^2+sigma^2] to take into account both variability and proximity to the target (precision and accuracy) by combining them together (Barone and Franco 2012). Particular attention was paid by operators to distinguish cultivated plants, perhaps abandoned, from those born spontaneously (Fig. 1). In addition, the total time spent was measured and the total cost estimated. The cost of the “by car” method was calculated, based on the average hourly salary of a researcher in Italy, for two researchers (driver and recorder) and the average mileage reimbursement in Italy in 2020 according to the ACI (Automobile Club of Italy) tables (http://www.aci.it). The cost of the ”by foot”, “GSV interpretation” and “Google Earth aerial view” methods only takes into account the average hourly cost of a researcher’s salary in Italy. "By foot" and "by car" costs are underestimated because they still require considerable resources (the time needed to travel to the study area, accommodation for longer campaigns etc.).

A ranking of the methods was determined by calculating a total score for each method. By assigning a relative weight of importance to each method quality feature and giving a relative score to each of them, based on the real outcome of the pilot experiment, the highest total score was found (Barone and Franco 2012). Higher relative weights of importance for the experiment were assigned to the parameters "percentage of identified taxa", "time" and "monetary cost".

#### Survey on the whole Sicily using GSV panoramas interpretation

**Figure 2.**
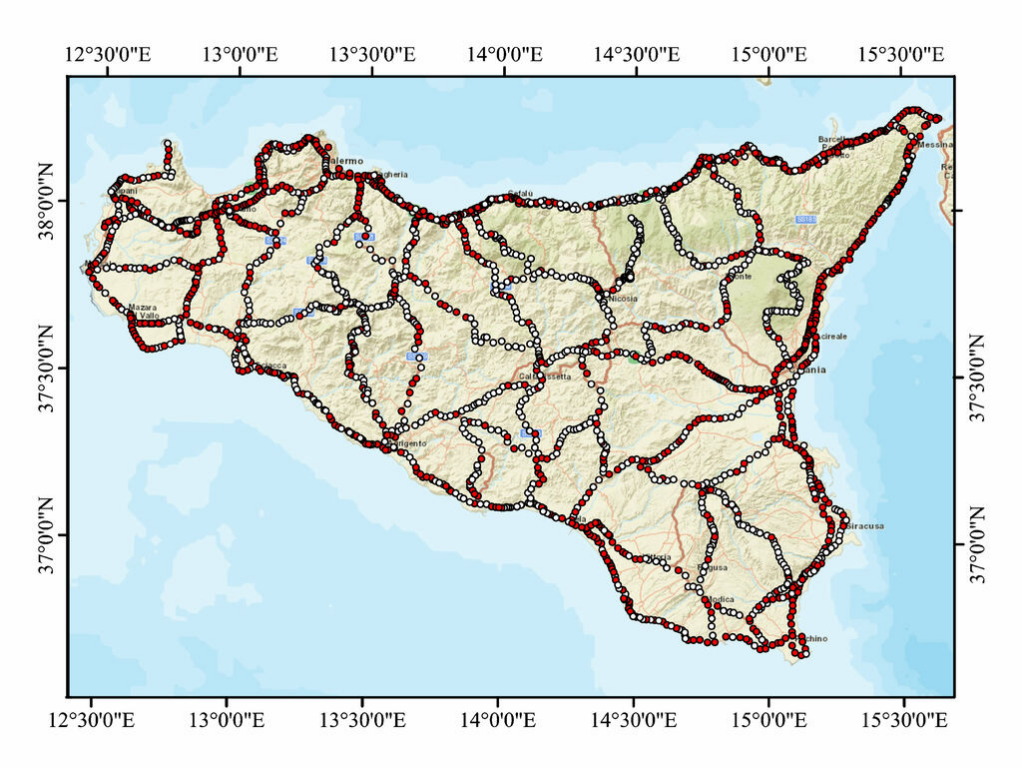
Distribution of the analysed GSV panoramas along the Sicilian road network: red) observations with aliens; white) observations without alien.

A survey on the entire Sicilian road network with GSV interpreting panoramas at 1.5 km from each other on all the highways and on a selection of the main and the local roads was done. The selection of main and local roads was done to cover the whole territory of the region with a homogeneous network (Fig. 2).

Overall, 2,350 panoramas were interpreted in 35 hours, along more than 3,500 km of roads. For each observation, the following data were recorded: coordinates in decimal degrees (WGS84), type of road (Highway, Main Road and Local Road), image capture date and occurrence of alien taxa. The altitude for each point was extracted from a digital terrain model of 2 × 2 m resolution (from http://www.sitr.regione.sicilia.it). Data concerning the taxa identified were added in a second stage: family, life form, origin according to Pignatti (2017), Pignatti (2018), archaeophytic or neophytic status, means of introduction, degree of naturalisation according to Galasso et al. (2018b) and main reproductive strategy adopted. In addition, data concerning the locality: the land use according to the Corine Land Cover classification (Kosztra and Buttner 2019) and protected areas (from http://www.sitr.regione.sicilia.it), the synthetic cartography units (according to Domina et al. 2018) and the bioclimate (according to Bazan et al. 2015).

The taxa identification was based on the skills of the authors, with the help of relevant local literature (Raimondo and Domina 2007, Cambria et al. 2015). The identification of seedlings was based on the presence of the mother plants in the location, when available (e.g. distinction between *Washingtonia robusta* H.Wendl. and *W. filifera* (André) de Bary). The results were compared with the whole alien flora of Sicily (Galasso et al. 2018a, Galasso et al. 2018b, Galasso et al. 2019a, Galasso et al. 2019b, Galasso et al. 2020).

## Results

### Comparison between different survey methods on the 10 km stretch of road

The relief on foot resulted in a list of 34 specific and subspecific taxa. The one by car and by GSV panoramas interpretation along the entire route resulted in the same 21 taxa. The relief interpretation of GSV panoramas, each of 1.5 km, resulted in 10 taxa and the interpretation of Google Earth aerial images resulted in five taxa only (Table 1).

The results obtained by car and from the complete observation of the entire route with GSV give better results with highly visible species (e.g. trees, shrubs, plants with large flowers etc.) than with inconspicuous species (annuals, hemicriptophytes, bulbs etc.) (Table 2). Their results are almost identical with a similar investment of time, but the overall cost of the GSV is significantly lower. The interpretation of GSV panoramas, each 1.5 km, consisting in the study of only seven panoramas, allowed us to record only 14 occurrences of the 10 identified taxa. The manual interpretation of aerial photos is the method that has given the worst results in terms of the number of species that can be identified, but has allowed us to record 80 occurrences of the five recognised species (Table 3). The expected loss gives the lower value for the GSV complete observation, followed by the "by car" method and with values 10 and 30 times higher than those of Google earth aerial and GSV, each of 1.5 km, respectively (Table 3). The calculated total score of the methods (Table 4) is higher for the complete interpretation of the GSV panoramas (0.8), the other three expeditious methods giving an equal score (0.7). The "by foot" method is the one that gave a lower score (0.4) due to the highest time and cost. Different rankings can be formulated by assigning different weights to the single parameters, based on the research objectives and the availability of resources.

### Survey on the whole of Sicily, using GSV panoramas interpretation

**Figure 3.**
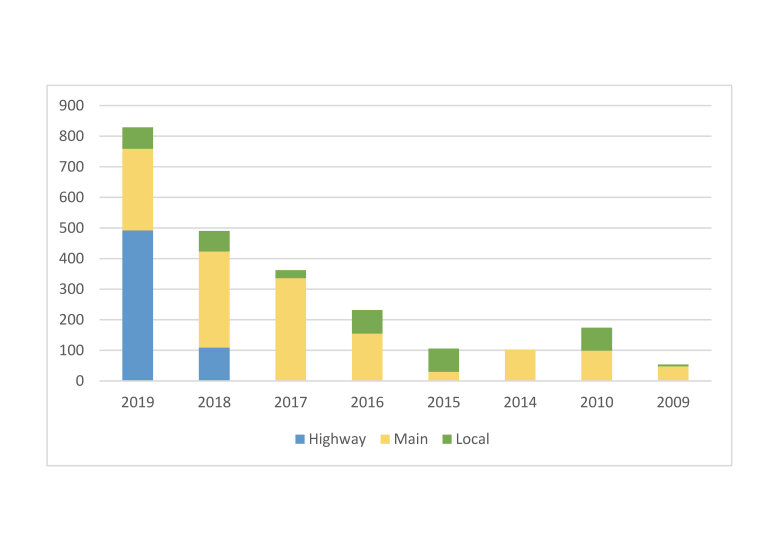
Number of interpreted GSV panoramas, divided by image capture date and road type.

The panoramic photos, used for this survey on the whole Sicily, date back to 2009-2019. However, 56%, including all highway shots, are not older than 3 years and 81% are not older than 5 years. (Fig. 3). Out of the 415 known aliens in Sicily, 394 grow along a road (unpublished data of the authors), but only 40 were identified in the 2,350 interpretations made. Concerning life forms: 22 taxa are Phanerophytes, 2 Chamaephytes, 3 Hemicryptophytes, 5 Geophytes, 4 Therophytes, 3 Nanophanerophytes and 1 Helophyte (Table 5). Almost half of the taxa recorded are native to the American continent, followed by African and Asian species and then all the other origins. The same ratio was recorded for the entire alien contingent on the Island (Raimondo et al. 2005).

The most commonly recorded species are: *Arundo donax* L. (396 occurrences), *Ailanthus altissima* (Mill.) Swingle (171), *Acacia saligna* (Labill.) H.L.Wendl. (170) and *Opuntia ficus-indica* (L.) Mill (133) (Table 6).

The taxa surveyed mostly are naturalised in Sicily (26 taxa), 12 are invasive and only 2 are casual (Table 7); most of them are neophytes (33) and 7 are archaeophytes (Suppl. material 1).

Four species were accidentally introduced, while 22 were introduced as ornamentals, 10 for forestry or for stabilisation of dunes or road embankments. Only *Opuntia ficus-indica* is a food plant, although its spread along the road network is, in part, also due to ornamental or fence purposes. *Agave sisalana* Perrine, *Arundo donax*, *Rhus coriaria* L. and *Ricinus communis* L. are species introduced into Sicily for industrial purposes, but, once their exploitation ended, they remained on the Island and spread.

The large number of taxa recorded reproduces by seeds (24), 12 reproduce both by seeds and vegetatively and four almost exclusively through vegetative reproduction (Suppl. material 1). Highways and Main Roads are more invaded than Local roads (Table 7). As expected, the areas with a higher presence of alien taxa are those that are strongly disturbed: mines (100% of presences), urban areas (66.3%), industrial areas (63.2%), permanent crops (61.4%) and pastures (60.1%). The areas with the lowest percentage are those with the least anthropogenic disturbance: forests (16.7%) and open spaces with little vegetation (29%); arable land (37.8%), on the contrary, is continuously disturbed. Protected areas show a lower percentage of observations with aliens in comparison with non-protected ones (Table 7); but, if we focus on protected areas near the coast, they are almost equally affected by alien species on roadsides as are non-protected areas.

**Figure 4.**
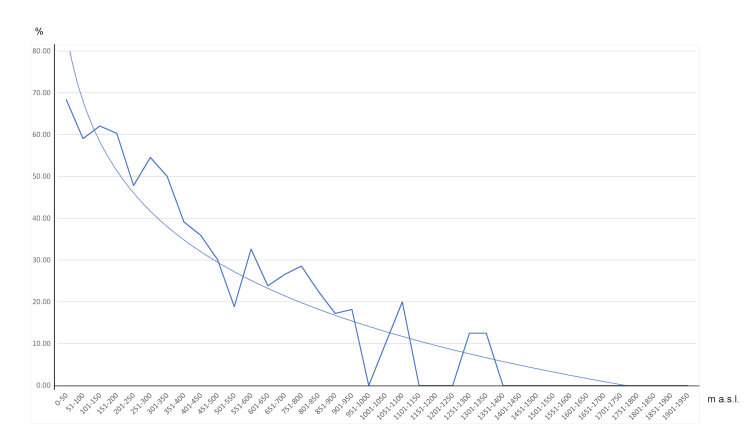
Percentage variation of photo-interpretations in which alien species were found with varying altitude.

Coasts and inland areas with Thermomediterranean climate have the highest percentage of alien taxa, whereas higher mountains with Mesomediterranean or Supramediterranean bioclimates have the lowest percentages of alien taxa (Fig. 4). The Eastern coast and the Peloritani Mountains are the units with higher percentages of observations with aliens.

## Discussion

The method that gave the best results in terms of number of species is the survey by foot. Taking into account all the other parameters considered, including cost and time, the photo-interpretation of GSV panoramas was found to be preferable for monitoring the occurrence of alien plants along the roads. This method showed some limitations compared to images provided by satellites. The former are updated no more than once per year (Tooth 2006) and are confined to the vicinity of roadways, the latter are updated frequently, often weekly and are spatially comprehensive and multispectral and allow us to measure the extent of plant populations even outside the immediate vicinity of the road. Manual interpretation of aerial images resulted in a limited number of taxa identified and do not trace the presence of single, young, individuals that, conversely, were mapped by car survey or using GSV.

Despite the limitations that emerged, we wanted to make a quick survey of the entire Sicilian road network, using the GSV panoramas interpretation every 1.5 km that gave the same score as the other expeditive methods tested. The study of the entire regional territory, using GSV images spaced each 1.5 km, recorded only 10% of the alien species known for Sicily. This is due to the limited territory overall observed and to the impossibility of identifying a large number of taxa from photos done from a moving car, such as those used for GSV shots. In addition, using GSV panoramas, it is not possible to choose the season in which to carry out the surveys (Tooth 2006). This makes identification impossible for many species that are evident during flowering, but little visible during the rest of the year, such as *Cenchrus setaceus* (Forssk.) Morrone or *Oxalis pes-caprae* L.

For the observed case study, it was recorded that, where the roads cross well-tended agricultural areas, the presence of alien species is very low. The annual tillage of the land tends to limit the presence of perennial alien species, with the sole exclusion of *Arundo donax* that was favoured in these contexts for the uses made of it which continue to the present day (Jiménez-Ruiz et al. 2021). Protected areas, especially coastal ones, are almost equally affected by alien species on roadsides as are non-protected areas. This is due the high degree of disturbance in these areas and to a lack of any management aimed at limiting the spread of these species. Forest remnants can limit the estblishment and dispersal of the light-demanding alien species such as *Acacia* sp. pl. (Heringer et al. 2020). On the contrary, the opening of roads leads to the modification of natural plant communities (Pollnac et al. 2012) which become susceptible to the entry of invasive alien species. In urban areas (Land use 1.1 in Table 7), there is a large incidence of alien species which includes a good percentage of plants that have escaped cultivation (Herrando-Moraira et al. 2020). Suburbs are subject to general disturbance which creates large spaces suitable for the most aggressive taxa (Domina et al. 2019, Szumańska et al. 2021). Along the highways, where generic vegetation cuts are made to ensure the clearing of carriageways and road edges, there is a smaller number of species, but a higher number of occurrences.

The interpretation of GSV photos can provide a minimal fraction of the species present in an area; however, in 35 hours, it was possible to carry out 2350 surveys distributed throughout the Sicilian road network which allowed us to map the distribution of widespread species, such as *Acacia saligna*, *Ailanthus altissima* and *Opuntia ficus-indica* species often neglected in floristic studies and herbaria (Williams and Lutterschmidt 2006) for which generic distributions were known. These data are the basis for drawing up monitoring and control plans for dangerous invasive species.

## Conclusions

Roadside surveys are a useful tool for compiling and updating alien plants inventories, especially with limited time availability and small inventory budget (Brundu et al. 2011, Christen and Matlack 2006). The interpretation of GSV panoramas resulted in a method suitable for monitoring the occurrence of alien plants along the roads, better than other expeditive methods as the survey by car of the aerial photo intepretation. With low costs, it is possible to explore large portions of the territory (Deus et al. 2016). The main limitations concern the fact that the images were acquired along road axes, therefore, they affect only a limited number of habitats. Continuous observation along the road or observations of panoramas at regular intervals can be planned. It is difficult to predict the usefulness in the future of semi-automatic systems for species recognition as tested by Ringland et al. (2019) for the recognition of crops, because spontaneous plants are highly variable compared to cultivated ones. Moreover, once the software recognises the morphological characters of the investigated species, the intervention of the operator would be fundamental to distinguish cultivated individuals from spontaneous ones. None of the compared remote sensing techniques is able, by itself, of giving the same results as the survey by foot, but by adopting a mix of techniques, it is possible to carry out surveys of large areas in shorter times and with lower costs than the traditional survey. Remote sensing techniques may be a useful tool to assist in habitat surveys and census of biodiversity, reducing also survey-related costs (e.g. transportation time and mileage, fossil fuel consumption (Olea and Mateo-Tomás 2013, Pearce et al. 2007).

The GSV sampling methodology, applied to the identification of alien plants on roadsides, can definitely act as a starting point for further field investigations and for developing management policies at a local level.

## Supplementary Material

1D8FAD58-2C24-5C40-86D9-584DB8FB10AD 10.3897/BDJ.9.e66013.suppl1 Supplementary material 1 Alien plants identified along the Sicilian Road Network Data type occurences File: oo_520798.csv https://binary.pensoft.net/file/520798 Giulio Barone, Gianniantonio Domina, Emilio Di Gristina

## Figures and Tables

**Table 1.** Taxa identified in the 10 km of road. The number of segments of 100 m in which each taxon was recorded on foot and mean of the number of segments by car, with continuous interpretation of GSV panoramas, interpreting a GSV each 1.5 km and with Google earth aerial view is indicated.

**Taxon**	**Life form**	**On foot**	**By car**	**GSV complete**	**GSV each 1.5 km**	**Google Earth**
*Acacia saligna* (Labill.) H.L.Wendl.	P scap	8	8	8	1	5
Agave americana L. subsp. americana	P caesp	1	1	1		
*Agave sisalana* Perrine	P caesp	2	2	2	1	1
*Ailanthus altissima* (Mill.) Swingle	P scap	6	5	5	1	
*Amaranthus retroflexus* L.	T scap	1				
*Arundo donax* L.	G rhiz	80	73	75	3	70
*Asclepias fruticosa* L.	P caesp	2	1	2		
*Asparagus setaceus* (Kunth) Jessop	G rhiz	2				
*Bidens pilosa* L.	T scap	4				
*Boerhavia coccinea* Mill.	T scap	8	6	6		
*Cascabela thevetia* (L.) Lippold	P scap	1				
*Cenchrus setaceus* (Forssk.) Morrone	H caesp	3				
*Erigeron bonariensis* L.	T scap	7	1	1	1	
*Erigeron canadensis* L.	T scap	4	2	2		
*Ipomoea indica* (Burm.) Merr.	G rhiz	7	7	7	1	
Lantana camara subsp. aculeata (L.) R.W.Sanders	P caesp	2	2	2		
Leucaena leucocephala subsp. glabrata (Rose) Zárate	P scap	2	2	2		
*Medicago sativa* L.	H scap	1				
*Melia azedarach* L.	P scap	1	1	1		
*Mirabilis jalapa* L.	G bulb	1	1			
*Opuntia ficus-indica* (L.) Mill.	P succ	11	10	11	1	
*Oxalis pes-caprae* L.	G bulb	1	1			
*Eryobotrya japonica* (Thunb.) Lindley	P scap	1				
*Ricinus communis* L.	P scap	53	49	49	2	
*Robinia pseudoacacia* L.	P scap	7	7	7	1	2
*Saccharum biflorum* Forssk.	H caesp	4	4	4		2
*Setaria adhaerens* (Forssk.) Chiov.	T scap	1				
*Sorghum halepense* (L.) Pers.	G rhiz	15	12	12	2	
*Symphyotrichum squamatum* (Spreng.) G.L.Nesom	T scap	10	5	5		
*Tropaeolum majus* L.	T rept	1				
*Vachellia karroo* (Hayne) Banfi & Galasso	P scap	3	3	3		
*Vitis × ruggerii* Ardenghi, Galasso, Banfi & Lastrucci	P lian	1	1	1		
*Washingtonia robusta* H.Wendl.	P scap	2				
*Xanthium italicum* Moretti	T scap	5	3	2		
**TOTAL**		258	207	208	14	80

**Table 2.** Recorded life forms in 10 km using the different methods. The percentages are calculated with respect to the value of the "on foot" survey. Ch: Chamaephyte, G: Geophyte, H: Hemicryptophyte, He: Helophyte, NP: Nanophanerophyte, P: Phanerophyte, T: Therophytes, bienne: biannual, bulb: bulbose, caesp: caespitose, lian: lianoid, rhiz: rhizomatous, scap: scapose, succ: succulent, suffr: suffruticose.

	**On foot**		**By car**		**GSV complete**		**GSV each 1.5 km**		**Google Earth aerial**	
	**No.**	%	**No.**	%	**No.**	%	**No.**	%	**No.**	%
**P scap**	84	100.00	75	89.29	75	89.29	5	5.95	7	8.33
**P caesp**	7	100.00	6	85.71	7	100.00	1	14.29	1	14.29
**P lian**	1	100.00	1	100.00	1	100.00	0	0	0	0
**P succ**	11	100.00	10	90.91	11	100.00	1	9.09	0	0
**H scap**	1	100.00	0	0	0	0	0	0	0	0
**H caesp**	7	100.00	4	57.14	4	57.14	0	0	2	28.57
**G bulb**	2	100.00	2	100.00	0	0	0	0	0	0
**G rhiz**	104	100.00	92	88.46	94	90.38	6	5.77	70	67.31
**T scap**	40	100.00	17	42.50	16	40.00	1	2.50	0	0
**T rept**	1	100.00	0	0.00	0	0.00	0	0.00	0	0
**TOTAL**	258	100.00	207	80.23	208	80.62	14	5.43	80	31.01

**Table 3.** Comparison between the five tested methods. Taxa identified and time spent are measured. The cost is estimated and rounded to the nearest unit.

**Parameter**	**By foot**	**By car**	**GSV complete**	**GSV each** **1.5 km**	**Google Earth aerial**
**Viewshed**	100%	90%	90%	50%	90%
**Taxa identified (No.; %)**	34; 100%	21; 62%	21; 62%	10; 29%	5; 15%
**Expected loss**	-	340.42	178.03	9268	3702.08
**Mapping of single individuals**	yes	yes	yes	yes	only for trees and shrubs
**Choice of the season**	yes	yes	no	no	no
**Weather influence**	yes	yes	no	no	no
**Possibility of frequent updates**	yes	yes	no	no	no
**Measurement of plant population extension**	no	no	no	no	yes
**Safety for the researcher**	medium / low	medium	high	high	high
**Access to highways**	no	yes	yes	yes	yes
**Time per 10 km (min)**	160	17	15	5	10
**Cost (€)**	26	15	3	1	2

**Table 4.** Comparison between the five tested methods. Relative scores to each method are based on the real outcome of the pilot experiment.

**Parameter**	**Relative weight of importance**	**By foot**	**By car**	**GSV complete**	**GSV** **each** **1.5 km**	**Google Earth aerial**
**Viewshed**	0.044	1.00	0.90	0.90	0.50	0.90
**Taxa identified**	0.2	1.00	0.62	0.62	0.29	0.15
**Expected loss**	0.044	1.00	0.96	0.98	0.00	0.60
**Mapping of single individuals**	0.044	1.00	1.00	1.00	1.00	0.50
**Choice of the season**	0.044	1.00	1.00	0.00	0.00	0.00
**Weather influence**	0.044	0.00	0.00	1.00	1.00	1.00
**Possibility of frequent updates**	0.044	1.00	1.00	0.00	0.00	0.00
**Measurement of plant population extension**	0.044	0.25	0,25	0.25	0.25	1.00
**Safety for the researcher**	0.044	0.25	0.50	1.00	1.00	1.00
**Access to highways**	0.044	0.00	1,00	1.00	1.00	1.00
**Time per 10 km**	0.2	0.00	0.89	0.91	0.97	0.94
**Cost**	0.2	0.00	0.42	0.88	0.96	0.92
**Total**	1	**0.4**	**0.7**	**0.8**	**0.7**	**0.7**

**Table 5.** Biological and chorological spectra of the investigated florula on the Sicilian road network.

**Life form**	**No. taxa**	%		**Origin**	**No. taxa**	%
P scap	13	32.5		America	19	47.5
P caesp	4	10		Africa	5	12.5
P succ	3	7.5		Asia	5	12.5
P lian	2	5		Australia	3	7.5
NP	3	7.5		Canary Is.	2	5
Ch suffr	2	5		Europe	2	5
H caesp	2	5		Paleotrop.	2	5
H bienne	1	2.5		Europe. Asia	1	2.5
G bulb	2	5		Madagascar	1	2.5
G rhiz	3	7.5		**Total**	40	
He	1	2.5				
T scap	4	10				
**Total**	40					

**Table 6.** Taxa identified along the whole Sicilian Road Network using GSV panoramas interpretation, each of 1.5 km.

**Taxon**	**No. of occurrences**
*Arundo donax* L.	396
*Ailanthus altissima* (Mill.) Swingle	171
*Acacia saligna* (Labill.) H.L.Wendl.	170
*Opuntia ficus-indica* (L.) Mill.	133
*Ricinus communis* L.	68
*Rhus coriaria* L.	46
*Cenchrus setaceus* (Forssk.) Morrone	42
*Robinia pseudoacacia* L.	36
Agave americana L. subsp. americana	29
*Oxalis pes-caprae* L.	23
*Symphyotrichum squamatum* (Spreng.) G.L.Nesom	19
*Ipomoea indica* (Burm.) Merr.	17
*Saccharum biflorum* Forssk.	12
*Phoenix canariensis* H.Wildpret	11
*Agave sisalana* Perrine	7
*Erigeron canadensis* L.	7
*Vachellia karroo* (Hayne) Banfi & Galasso	7
*Erigeron bonariensis* L.	4
*Carpobrotus acinaciformis* (L.) L.Bolus	3
Cyperus alternifolius subsp. flabelliformis Kük.	3
Lantana camara subsp. aculeata (L.) R.W.Sanders	3
*Mirabilis jalapa* L.	3
*Washingtonia robusta* H.Wendl.	3
*Cardiospermum grandiflorum* Sw.	2
Eucalyptus camaldulensis Dehnh. subsp. camaldulensis	2
Isatis tinctoria L. subsp. tinctoria	2
Leucaena leucocephala subsp. glabrata (Rose) Zárate	2
*Senecio angulatus* L.f.	2
*Aeonium arboreum* (L.) Webb & Berthel.	1
*Alnus cordata* (Loisel.) Duby	1
*Austrocylindropuntia cylindrica* (Lam.) Backeb.	1
*Canna indica* L.	1
*Melia azedarach* L.	1
*Mesembryanthemum cordifolium* L.f.	1
*Opuntia stricta* (Haw.) Haw.	1
*Parasenegalia visco* (Griseb.) Seigler & Ebinger	1
*Parkinsonia aculeata* L.	1
*Solanum elaeagnifolium* Cav.	1
*Solanum lanceolatum* Cav.	1
*Xanthium italicum* Moretti	1
Total	1235

**Table 7.** Invasive, naturalised and casual taxa per type of road, altitudinal range, cartographic unit, protected area, bioclimate and land use. Using GSV, each of 1.5 km, on the whole Sicilian road network.

**Type of road**	**Obs.**	**Obs. with aliens**	%	**Invasive**	%	**Naturalised**	%	**Casual**	%
Highway	601	393	65.4	323	82.2	70	17.8	0	0.0
Main	1352	676	50.0	515	76.2	159	23.5	2	0.3
Local	397	166	41.8	129	77.7	37	22.3	0	0.0
**Total**	2350	1235	52.6	968	78.4	266	21.5	2	0.2
**Cartographic unit**	**Obs.**	**Obs. with aliens**	%	**Invasive**	%	**Naturalised**	%	**Casual**	%
2.1 Northern coast	329	222	67.5	181	81.5	41	18.5	0	0
2.2 Eastern coast	138	107	77.5	78	72.9	29	27.1	0	0
2.3 Southern and Western coast	144	100	69.4	76	76.0	24	24.0	0	0
3.1 Western Sicily and inland Palermo	507	300	59.2	256	85.3	42	14.0	2	0.7
3.2 Hilly inland	507	221	43.6	159	71.9	62	28.1	0	0
4.2 Mts of Palermo	23	8	34.8	6	75.0	2	25.0	0	0
4.3 Sicani Mts	43	10	23.3	2	20.0	8	80.0	0	0
4.4 Madonie Mts	62	16	25.8	13	81.3	3	18.8	0	0
4.5 Erei Mts	77	18	23.4	15	83.3	3	16.7	0	0
4.6 Nebrodi Mts	121	30	24.8	21	70.0	9	30.0	0	0
4.7 Peloritani Mts	29	21	72.4	17	81.0	4	19.0	0	0
4.8 Etna Mt.	162	88	54.3	76	86.4	12	13.6	0	0
4.9 Iblei and Siracusa Mts	208	94	45.2	67	71.3	27	28.7	0	0
**Altitude m a.s.l.**	**Obs.**	**Obs. with aliens**	%	**Invasive**	%	**Naturalised**	%	**Casual**	%
0-300. Plain	1607	1026	63.8	813	79.2	211	20.6	2	0.2
301-600. Hill	381	141	37.0	102	72.3	39	27.7	0	0.0
601-2000. Mountain	362	68	18.8	52	76.5	16	23.5	0	0.0
**Biolclimate**	**Obs.**	**Obs. with aliens**	%	**Invasive**	%	**Naturalised**	%	**Casual**	%
1. Lower Thermomediterranean	1069	701	65.6	562	80.2	137	19.5	2	0.3
2. Upper Thermomediterranean	608	374	61.5	289	77.3	85	22.7	0	0
3. Lower Mesomediterranean	408	119	29.2	88	73.9	31	26.1	0	0
4. Upper Mesomediterranean	185	35	18.9	23	65.7	12	34.3	0	0
5. Supramediterranean	80	6	7.5	5	83.3	1	16.7	0	0
**Land use**	**Obs.**	**Obs. with aliens**	%	**Invasive**	%	**Naturalised**	%	**Casual**	%
1.1 Urban fabric	285	189	66.3	147	77.8	42	22.2	0	0
1.2 Industrial- commercial and transport units	19	12	63.2	9	75.0	3	25.0	0	0
1.3 Mine- dump and construction sites	4	4	100.0	3	75.0	1	25.0	0	0
1.4 Artificial- non-agricultural vegetated areas	3	0	0.0	0	0	0	0	0	0
2.1 Arable land	564	213	37.8	168	78.9	45	21.1	0	0
2.2 Permanent crops	900	553	61.4	437	79.0	115	20.8	1	0.2
2.3 Pastures	276	166	60.1	133	80.1	32	19.3	1	0.6
3.1 Forests	90	15	16.7	7	46.7	8	53.3	0	0
3.2 Scrub or herbaceous vegetation associations	177	74	41.8	55	74.3	19	25.7	0	0
3.3 Open spaces with little or no vegetation	31	9	29.0	7	77.8	2	22.2	0	0
5.1 Inland waters	1	0	0.0	0	0	0	0	0	0
**Type of area**	**Obs.**	**Obs. with aliens**	%	**Invasive**	%	**Naturalised**	%	**Casual**	%
Protected area	249	101	40.6	69	68.3	32	31.7	0	0
Non-protected area	2101	1134	54.0	898	79.2	234	20.6	2	0.2
